# Novel Acrylamide/2-Acrylamide-2-3 Methylpropanesulfonic Acid/Styrene/Maleic Anhydride Polymer-Based CaCO_3_ Nanoparticles to Improve the Filtration of Water-Based Drilling Fluids at High Temperature

**DOI:** 10.3390/gels8050322

**Published:** 2022-05-20

**Authors:** Zhichuan Tang, Zhengsong Qiu, Hanyi Zhong, Hui Mao, Kai Shan, Yujie Kang

**Affiliations:** 1 School of Petroleum Engineering, China University of Petroleum (East China), No. 66 Changjiang West Road, Economic & Technical Development Zone, Qingdao 266580, China; b17020063@s.upc.edu.cn (Z.T.); zhonghanyi@126.com (H.Z.); b20020005@s.upc.edu.cn (K.S.); 17854210608@163.com (Y.K.); 2 State Key Laboratory of Oil & Gas Reservoir, Chengdu University of Technology, 1 East 3 Road, Chengdu 610059, China; maohui17@cdut.edu.cn

**Keywords:** filtration reducer, high temperature, water-based drilling fluid, nanomaterials, calcium carbonate

## Abstract

Filtration loss control under high-temperature conditions is a worldwide issue among water-based drilling fluids (WBDFs). A core–shell high-temperature filter reducer (PAASM-CaCO_3_) that combines organic macromolecules with inorganic nanomaterials was developed by combining acrylamide (AM), 2-acrylamide-2-methylpropane sulfonic acid (AMPS), styrene (St), and maleic anhydride (MA) as monomers and nano-calcium carbonate (NCC). The molecular structure of PAASM-CaCO_3_ was characterized. The average molecular weight of the organic part was 6.98 × 10^5^ and the thermal decomposition temperature was about 300 °C. PAASM-CaCO_3_ had a better high-temperature resistance. The rheological properties and filtration performance of drilling fluids treated with PAASM-CaCO_3_ were stable before and after aging at 200 °C/16 h, and the effect of filtration control was better than that of commonly used filter reducers. PAASM-CaCO_3_ improved colloidal stability and mud cake quality at high temperatures.

## 1. Introduction

With the deep exploration and development of oil and gas resources, high-temperature water-based drilling fluid (WBDF) technology has become one of the key technologies in drilling engineering [1,2,3]. As the most commonly used high-temperature drilling fluid additive for a long time, additive materials (such as sulfonated phenolic resin, sulfonated lignite, sulfonated tannin) have been widely used to control rheology or filtration performance [4,5,6,7]. However, sulfonated materials still decompose easily at high temperatures [8], and some need to be used together to achieve the best results [9], most of which are environmentally unfriendly [10,11]. In view of the above disadvantages, since the 1980s, researchers have started to develop the application of multicomponent copolymers in high-temperature drilling fluids and have achieved good results [12,13,14].

As early as the 1980s, Giddings et al. [15] developed a terpolymer filtrate reducer. This agent was copolymerized with acrylamide, 2-acrylamide-2-methylpropanesulfonic acid, and 2-mercaptobenzoic acid as monomers. The rigid side chain and the large number of sulfonic acid groups in its molecule improve its high-temperature effect. Dickert et al. [16] developed a pH-adaptive high-temperature filtrate reducer by aqueous solution polymerization with acrylamide, 2-acrylamide-2-methylpropanesulfonic acid, and n-vinyl-alkyl amide as monomers. On the basis of Giddings, American scholar Patel [17] successfully prepared a high-temperature and high-salinity filtration reducer with 2-acrylamide-2-methylpropionic acid as the monomer and 2-mercaptobenzoic acid as the cross-linking agent based on precise control of the molecular structure. The agent can resist Ca^2+^ and Mg^2+^ ion pollution and has a good effect against temperatures exceeding 200 °C.

As the research progressed, the shortcomings of the descending agents of the polymers formed by copolymerization of AM monomers with alkene monomers and sulfonic acid monomers were gradually exposed [18,19,20]. Therefore, researchers began to develop environmentally friendly high-temperature and -salinity WBDF loss reducers on the basis of ethylene sulfonic acid monomer/acrylamide (or its derivatives). Thaemlitz et al. [21] developed a fluid loss reducer with high-temperature resistance and high salinity with N-vinyl carbazole (NVC), polystyrene sulfonic acid (PSS), and AMPS as monomers. Drilling fluids using it as a key additive can maintain good rheology and filtration after aging at high temperatures, and also have good anti-pollution and some shale inhibition effects.

In 2019, Soric and Heier [22] developed a high-temperature and high-salinity fluid loss reducer by aqueous solution polymerization. Its relative molecular weight was about 1 million and its temperature resistance exceeded 180 °C. The drilling fluid system constructed with it as a key treatment agent has been successfully applied to shale gas blocks in the Republic of Herwazka and exhibits a reservoir protection effect. The Exxon company has prepared an environment-friendly, high-temperature, and high-salinity WBDF system with synthetic polymer as the key treatment agent, which can resist the high temperature of 210 °C, and the waste drilling fluid can be directly discharged into the sea after being tested by the U.S. Environmental Protection Agency (EPA); Schlumberger also developed drilling fluid systems with a density of 2.20 g/cm^3^ and temperature resistance exceeding 220 °C. It has been used in some sensitive areas such as marine blocks in the United States and is environmentally friendly. Bagum et al. used aloe additive to form four representative drilling fluid formulations along the base bentonite. A complete rheological test and filtration test of mud additives with different concentrations were carried out to study the feasibility of this new additive.

The team of Prof. Zhengsong Qiu at the University of Petroleum, China, thoroughly studied the mechanism of action of high-temperature, high-density, and high-salinity drilling fluids and developed a HTP-1 filtration reducer using amps, NVP, DAAC as monomers [23]. It worked well at 240 °C with a NaCl content of 20%wt and a density of 2.0 g/cm^3^. Based on this, a novel filtrate reducer, FLR-1, was developed by introducing nanotechnology [24]. Its filtration loss effect is significant, and the HTHP filtration at 200 °C/16 h is only 20.5 mL. In addition, FLR-1 can also significantly improve the rheological properties of the drilling fluid system with excellent salt tolerance and meet the environmental protection standards. Researchers [25] have shown that nanomaterials can significantly improve the performance of drilling fluids and broaden their service conditions. Scientists have [26] applied zinc oxide nanoparticles prepared in the laboratory to WBDF. The results show that nanoparticles improve the rheological properties of WBDF. Adding a single nanomaterial to the WBDF will not significantly affect the API filtration volume. However, the mud cake thickness decreases with the concentration of nanoparticles. The results show that nanoparticles can improve the rheological properties. The application of waste nanomaterials [27,28] was summarized in rheological and lubricity testing, adequate rheological and filtration checks were performed on water-based drilling fluids, and the effect of waste as an additive was evaluated on drilling fluid performance. Minakov et al. [11] found that the yield stress and consistency index of nanoparticle drilling fluids increase with temperature. As particle size increases, their influence on the temperature dependence of drilling fluid viscosity increases. The addition of nanoparticles stabilizes the viscosity of drilling fluids relative to temperature.

In summary, great progress has been made in WBDF filtration reducer technology at high temperature in recent years, but the problems still exist [29,30]. At present, some vinyl copolymer drilling fluid reducing agents are nontoxic and environmentally friendly [31,32], but are easily (partially) degraded under high-temperature conditions [33,34]. Some of the degraded products may be toxic/lowly toxic, which will affect their eco-friendly performance [35,36,37].

Recent studies have shown [38,39,40] that nanoparticles have the advantages of high surface energy, thermal stability, and rigidity. By assembling block copolymers into the grid holes formed by polymer frameworks, the rigidity and thermal stability of nanomaterials can be combined with the advantages of salt resistance and toughness of polymers [41], thus further improving the high-temperature stability of modifiers, which also provides a new idea for the development of high-temperature WBDF agents.

Therefore, this paper summarizes the design concept of the molecular structure of the high-temperature WBDF filtration reducer (PAASM-CaCO_3_), which has excellent properties. The rigidity and thermal stability of inorganic nanomaterials were combined, the nanoparticles were embedded into the grid pores formed by the polymer framework, and the organic–inorganic nanocomposites with excellent properties were developed.

## 2. Materials and Methods

### 2.1. Materials

The main reagents for the reaction are detailed in Table 1.

In addition, common filter reducer products were purchased to compare PAASM-CaCO_3_ with PAASM-CaCO_3_ from Shida Innovative Technology Co., Ltd., Dongying, China: Driscal D and D-4; high-temperature polymer filtration reducer, 80A51; high-temperature calcium-resistant fluid loss reducer, jt888; high-temperature salt-resistant filtration reducer, PJA-2; bitumen filtration reducer, FT-A; sulfonated phenolic resin, SMP-1.

### 2.2. Methods

#### 2.2.1. Synthesis of Poly (AM-AMPS-St-MA)-CaCO_3_

**Figure 1:**
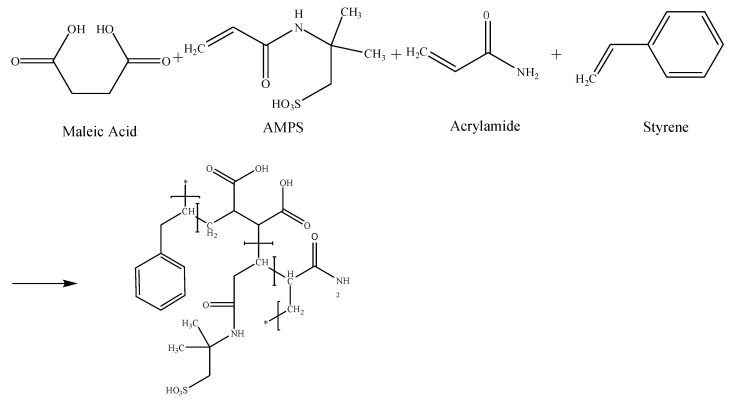
Schematic of Acrylamide/2-Acrylamide-2-methylpropanesulfonic Acid/Styrene/Maleic anhydride polymer (PAASM) synthesis.

**Figure 2:**
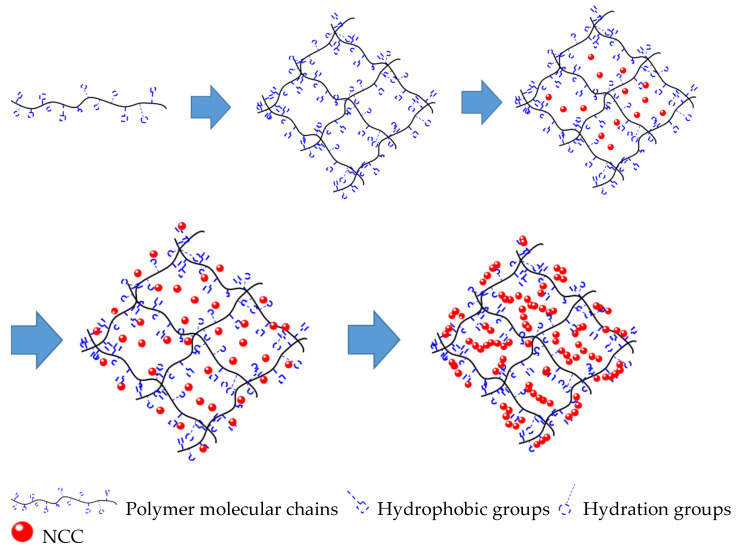
Schematic diagram of PAASM-CaCO_3_ reaction.

According to the designed molecular structure, monomers such as acrylamide (AM), maleic anhydride (MA), 2-acrylamide-2-methylpropanesulfonic acid (AMPS), and styrene (St) were used to prepare the high-temperature-resistant molecular framework, and then the pre-dispersed inorganic nanoparticles (NCC) were embedded into the prepared framework to ensure good temperature resistance. The reaction schematic of poly (AM-AMPS-St-MA)-CaCO_3_ (PAASM-CaCO_3_) is shown in Figure 1 and Figure 2.

An amount of 30 mL of sodium tetraborate buffer solution was prepared in beaker 1, and then 17.5 g of NCC was added. After high-speed stirring at 12,000 rpm for 15 min, 5 mL of dimethyl sulfoxide was added dropwise and placed in an ultrasonic cell disintegrator for dispersion. An amount of 50 mL of deionized water was added in beaker 2, and then 43.5 g of AMPS was added to dissolve with sufficient stirring. In beaker 3, 20 mL of deionized water was added and 29.8 g of AM was dissolved with stirring under heating. White oil, 1.25 g of tween 60, and 3.75 g of span 80 were added to beaker 4 and stirred well to homogeneity with a glass rod. The solutions in beaker 2 and beaker 3 were poured into a four-port flask, the pH values were adjusted to 6.0 with 15 mol/L of NaOH solution, 10.3 g of MA and 21.8 g of St were added, and the reaction system was stirred under the protection of N_2_. Beaker 2 and beaker 3 were cleaned with deionized water and added to a four-port flask. The liquid in beaker 4 was added into the flask and stirred for 15 min. Then, 0.336 g of K_2_S_2_O_8_ and NaHSO_3_ was added in beaker 5, dissolved by deionized water, and then added dropwise to the four-port flask. The reaction system began to heat up. The suspension in beaker 1 was dripped into the reaction system after 2 h and then continued to react for 5 h. Then, it was rapidly cooled to room temperature. Ethanol and acetone were added to filter the resulting sediment and quickly washed out with dilute hydrochloric acid. A novel polymer-based NCC with a core–shell structure as a high-temperature filtrate reducer for drilling fluid (PAASM-CaCO_3_) was obtained.

#### 2.2.2. Characterization of PAASM-CaCO_3_

(1) FTIR: 1 mg of dry PAASM-CaCO_3_ powder and 20 mg of KBr were mixed fully. The mixture was loaded into the mold and compacted with 50 MPa of pressure. FTIR spectra of the compacted tableting were obtained on a NEXUS FTIR spectrometer.
(2) TGA: The thermogravimetric analysis of PAASM-CaCO_3_ was carried out by a Mettler Teledo thermogravimetric analyzer in Switzerland. The temperature range was from room temperature to 1000 °C, the heating rate was 10 K/min, the atmosphere was nitrogen, and the gas flow rate was 50 mL/min.
(3) GPC: The relative molecular mass of PAASM (without NCC) was determined by the German SFD gel permeation chromatograph (GPC). The mobile phase was phosphate-buffered solution. The column was a SHODEX (K-806 M chloroform system) and the filler was styrene and two vinyl benzene copolymers.
(4) Surface hydroxyl number test: 18 mL of sodium dihydrogen phosphate-buffered solution was prepared, and then 12 mL of NaCl solution with a mass fraction of 0.2% was added.

After full mixing, the pH value of 0.5 mol/L of dilute hydrochloric acid sodium dihydrogen phosphate-buffered solution was adjusted to 5.5. The 0.6 g sample was fully stirred and dispersed, and the pH value of the liquid was measured with a precision pH meter. The suspension was titrated dropwise by a 1.5 mol/L NaOH solution to pH 9.0 for 20 s, which was the end point of the titration. The amount of NaOH consumed during this period was recorded

The hydroxyl number N on the surface of NCC can be calculated according to formula (1):

(1) $$N={\mathrm{CVN}}_{A}10^{-3}/\mathrm{Sm}$$

where C is the concentration of NaOH solution, mol/L; V is the amount of NaOH used from the start of the titration to the end point, mL; N_A_ is the Avogadro constant, 6.02 × 10^23^; S is the specific surface area of the particles, nm^2^/g; m is the mass of sample involved in the titration, g.

#### 2.2.3. Performance Evaluation of PAASM-CaCO_3_

(1) Drilling fluid preparation and aging

An amount of 16 g of sodium-based bentonite was added to 400 mL of clear water and stirred at 8000 rpm on a high-speed blender for 30 min, and then 0.8 g of Na_2_CO_3_ was added, stirred for 20 min, and then pre-hydrated for 24 h. A certain amount of polymer was then added to the fluid, which was stirred at 8000 rpm for 30 min on a high-speed blender. The composites were aged at a set temperature for 16 h by aging and were cooled to room temperature before stirring at high speed for 20 min. Rheological and filtration properties of drilling fluid before and after rolling at a specific temperature/16 h were tested according to the drilling fluid performance evaluation standard SY/t5621-1993.16 g.

(2) API Static Filtration Test

The static API filterability of drilling fluid was tested with a ZNZ-D3 API medium pressure filter (Qingdao Haitong Instrument Co., Ltd.). A certain amount of drilling fluid was loaded into the filter kettle, the top was covered with API filter paper, and it was placed under 100 psi. The filtered volume (FL or FL_API_) of the drilling fluid was recorded for 30 min, which is recommended by the API.

(3) High-temperature high-pressure static filtration loss test

The static HTHP filterability of drilling fluid was tested with a GGS424A high-temperature and high-pressure static filter instrument (Qingdao Haitongda Instrument Co., Ltd., Qingdao, China). A certain amount of drilling fluid was loaded into the filter kettle, the top cover was covered with HTHP filter paper, the top cover was tightened, and the difference between the upper and lower pressure was 3.5 MPa. The test temperature was the hot roll temperature (the test temperature is 180 °C when the hot roll temperature is higher than 180 °C). The filtered volume (FL_HTHP_) of the drilling fluid was recorded for 30 min, which is recommended by the API.

(4) Rheological property test

The rheological parameters of the drilling fluid were tested according to the drilling fluid performance evaluation standard SY/T5621-1993. The apparent viscosity, plastic viscosity, and yield point of drilling fluid were measured with the ZNP-M7 6-speed rotating viscometer (Qingdao Haitongda Instrument Co., Ltd.). They measured the apparent viscosity, plastic viscosity, and yield point of drilling fluid with φ600 and φ300. The value of 300 was calculated according to the test program recommended by the API.

AV = φ600/2(2)

PV = φ600 − φ300(3)

YP = φ300 − φ600/2(4)

where:

AV is the apparent viscosity (mPa·s);
PV is the plastic viscosity (mPa·s);
YP is the yield point (Pa);
φ600 is the dial reading of the 6-speed rotational viscometer at 600 r/min (dia);
φ300 is the dial reading of the 6-speed rotational viscometer at 300 r/min (dia).

#### 2.2.4. Study of Filtrate Control Mechanism

(1) Zeta potential test

A Brookhaven zeta potential tester (Brookhaven instruments Ltd., New York, NY, USA) was used to test the zeta potential of drilling fluid before and after aging. An amount of 8 g of sodium montmorillonite was added to 400 mL of deionized water and placed on a magnetic stirrer for 24 h. Then, a certain amount of PAASM-CaCO_3_ was added and stirred for 24 h to ensure that the various components of the mixed material were mixed sufficiently. Then, the drilling fluid was placed at a certain temperature and rolled for 16 h. When tested, drilling fluids were equipped with a microprocessor unit that automatically calculates the electron mobility of particles and converts them into ζ Potential. The average of the three tests was taken as the zeta potential of the drilling fluid.

(2) Particle size distribution test

An amount of 8 g of Na montmorillonite in 400 mL of deionized water was added and placed on a magnetic stirrer to stir for 24 h; then, a certain amount of PAASM-CaCO_3_ was added, stirring was continued for 24 h to ensure the various components in the mixed material were fully mixed, and the particle size distribution of drilling fluid before and after aging was tested by a bettersize2000 laser particle size distribution instrument (Dandong baited Instrument Co., Ltd., Dandong, China).

## 3. Results and Discussion

### 3.1. Structural Characterizations

#### 3.1.1. Fourier Transfer Infrared (FTIR) Analysis

**Figure 3:**
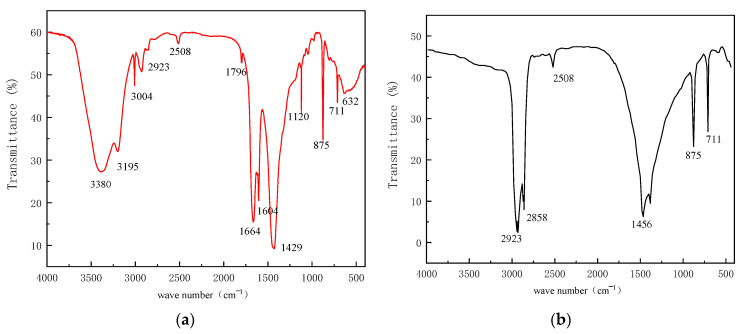
The FTIR analysis results. (**a**) PAASM-CaCO_3_; (**b**) NCC.

The results of the infrared spectroscopic analysis of PAASM-CaCO_3_ and NCC are shown in Figure 3. It can be seen from (a) that 3380 cm^−1^ is the O-H vibration absorption peak, 3195 cm^−1^ is the N-H stretching vibration absorption peak of the amide group, and 3004 cm^−1^ is the stretching vibration absorption peak. = C-H on the benzene ring, 2923 cm^−1^, is the C-H stretching vibration absorption peak on the saturated carbon atoms, 1796 cm^−1^ is the stretching vibration absorption peak of C=O in the carboxyl group; 1664 cm^−1^ can be attributed to the stretching vibration of C=O in the amide group; 1604 cm^−1^ is for the stretching vibration of the C=C skeleton of the benzene ring; 1429 cm^−1^ can be assigned to C-O antisymmetric stretching vibration; S=O symmetric contraction vibration in sulfonic acid groups appears at 1120 cm^−1^. The absorption peak at 875 cm^−1^ is assigned to the in-plane bending deformation vibration peak of CaCO_3_ C-O, and the absorption peak at 632 cm^−1^ is assigned to the in-plane deformation vibration peak of O-C-O, which indicates that the product contains NCC. There are no vibrational peaks of olefin double bonds at 1000 cm^−1^~900 cm^−1^, indicating no residual monomers in the products. The FTIR analysis result shows that the chains of the synthesized products have chains bearing all comonomers.

#### 3.1.2. Surface Hydroxyl Number Test

The results of the surface hydroxyl number test before and after NCC inlay copolymerization are shown in Table 2. From the test results, the number of surface hydroxyl groups of NCC is 0.1506/nm^2^ before modification, and the number of surface hydroxyl groups of PAASM-CaCO_3_ is drastically reduced to 0.0448/nm^2^ after modification, indicating that a large number of hydroxyl groups on the surface of NCC participate in the reaction and the size of the nanoparticles matches well with the size of the network structure.

#### 3.1.3. Gel Permeation Chromatography (GPC) Test

**Figure 4:**
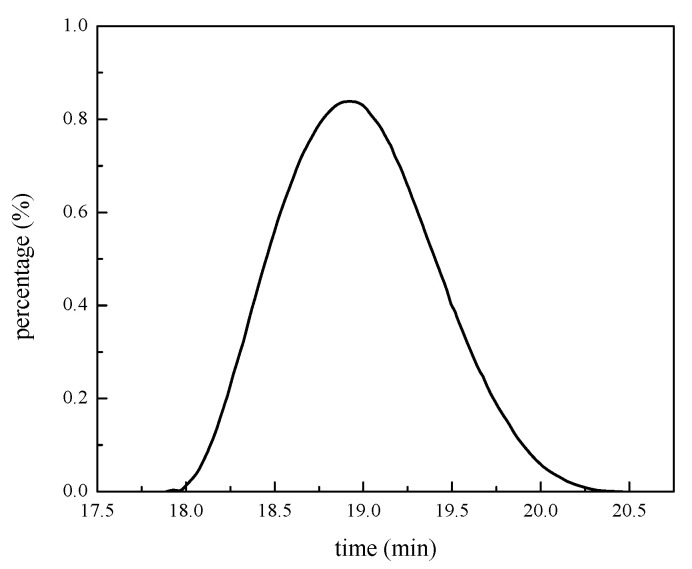
Results of GPC of PAASM.

The GPC experimental results of PAASM (without NCC) are shown in Table 3 and Figure 4. The results show that the weight-average molecular weight (Mw) of the polymer main chain is 6.98 × 10^5^, and the number-average molecular weight (Mn) is 2.84 × 10^5^, which gives PAASM a suitable relative molecular weight. It will be detrimental to the rheological regulation control of drilling fluid if the relative molecular weight of additive agents is too large. If the relative molecular weight is too small, it will be difficult to increase the viscosity, which will affect the effect of colloidal protection and filtration reduction, and it will be difficult to guarantee high-temperature stability. The relative molecular mass of PAASM is moderate and has the potential to overcome the above drawbacks. At the same time, it can be found that the relative molecular weight distribution of PAASM is narrow and the polydispersity coefficient is 2.45, which indicates that the molecular mass distribution of PAASM polymer is relatively uniform.

#### 3.1.4. Thermogravimetric Analysis

**Figure 5:**
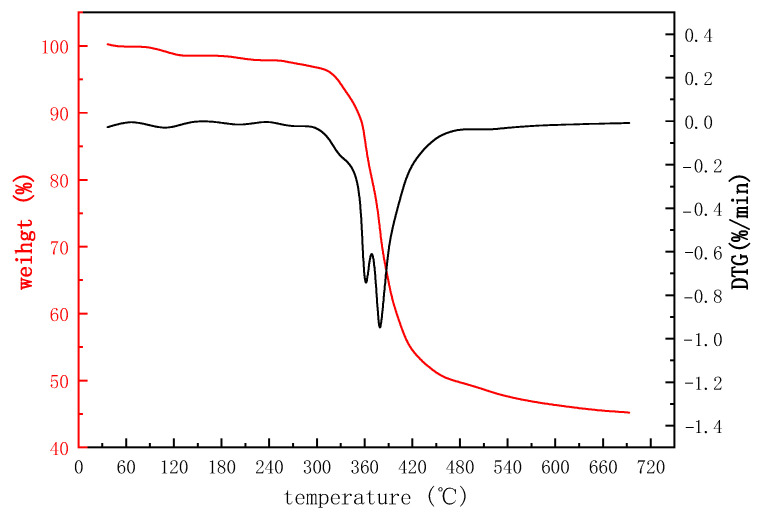
Thermogravimetric analysis results of PAASM-CaCO_3_.

The thermogravimetric analysis results of PAASM-CaCO_3_ are shown in Figure 5. As can be seen from Figure 5, there is no decomposition of PAASM-CaCO_3_ from room temperature to 300 °C, and the weight loss is mainly due to the re-removal of adsorbed water. The side chain begins to decompose from 330 °C to 420 °C. When the temperature is higher than 420 °C, the molecular skeleton is completely destroyed and the final residual mass is about 45%, which is mainly composed of NCC and the carbonized main chain structure. The results of thermogravimetric analysis show that the total thermal weight loss of PAASM-CaCO_3_ is about 55% in the range from room temperature to 500 °C. The thermal decomposition temperature of PAASM-CaCO_3_ is much higher than those of traditional agents.

#### 3.1.5. Micro-Morphology Test

**Figure 6:**
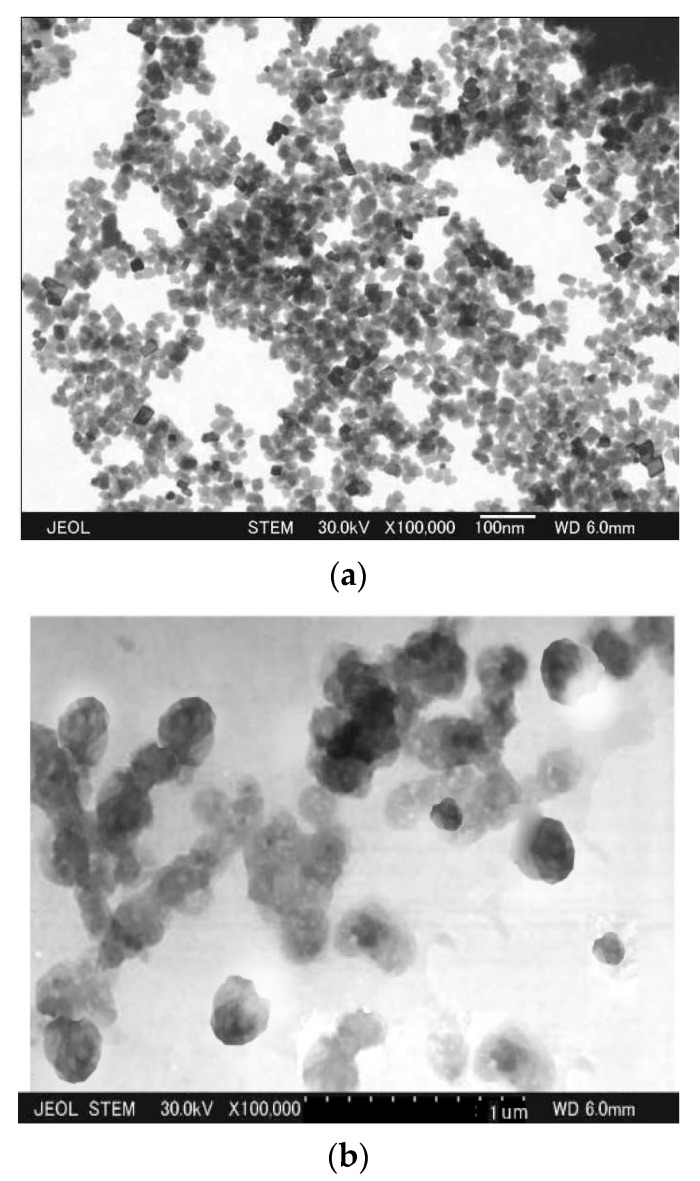
TEM test result. (**a**) NCC suspension; (**b**) PAASM-CaCO_3_ suspension.

TEM images of NCC and PAASM-CaCO_3_ in an aquatic environment are shown in Figure 6. It can be seen that the NCC before modification is cubic with an average particle size of about 15 nm (Figure 6a). The modified particles are spherical and the particle size changes to about 200 nm (Figure 6b). The reason for this change is that the modified polymer is coated on the surface of NCC, and the polymer swells in water and partially dissolves in water, resulting in adhesion, resulting in the increase in NCC particle size in TEM, which also indicates that NCC has been successfully modified.

**Figure 7:**
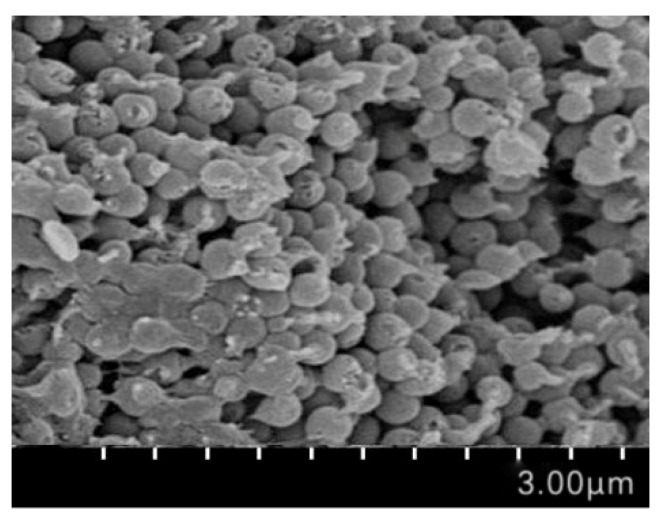
SEM photo of PAASM-CaCO_3_.

The SEM image of PAASM-CaCO_3_ is shown in Figure 7. It can be seen that PAASM-CaCO_3_ is mainly spherical with a large specific surface area, which can exert its surface energy advantage. The particles are closely packed, adhere to each other, and are basically connected by polymers. The particle size of the polymers is basically the same as that measured by TEM.

#### 3.1.6. Particle Size Distribution Test

**Figure 8:**
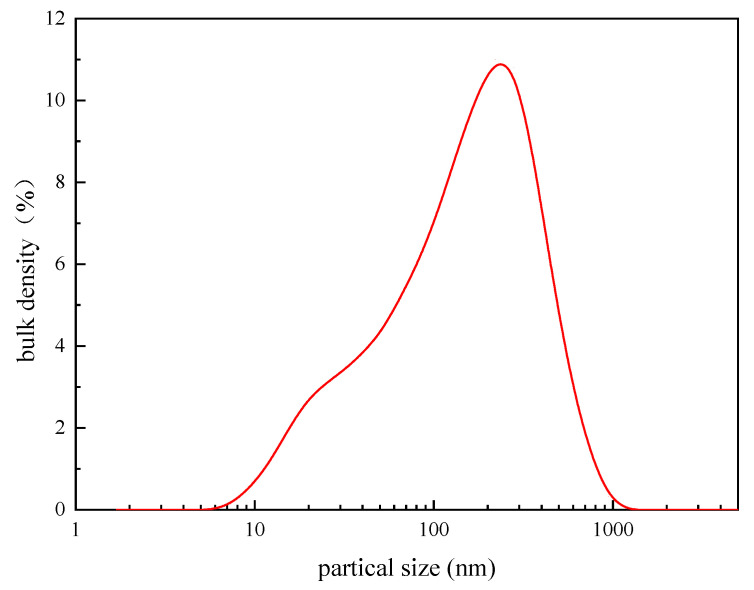
PAASM-CaCO_3_ particle size distribution.

The results of the particle size distribution of 0.1% PAASM-CaCO_3_ in water are shown in Figure 8. As can be seen from the test results, the PAASM-CaCO_3_ particle size distribution is narrow, with a D50 of approximately 259 nm, D10 of 1.88 nm, and D90 of 877 nm. The particle size test results are relatively close to the TEM results. Compared with the SEM results, the reason for the increase in particle size is mainly due to the water absorption of the polymer and the hydration in the solution.

### 3.2. Performance Evaluation

#### 3.2.1. Filtration Reduction Effect in 4% Bentonite Mud

The filtration volume under medium pressure and HTHP (200 °C/3.5 MPa) of PAASM-CaCO_3_ and commonly used high-temperature-resistant polymer filter reducers (coded Driscal-D and D-4) after aging at 200 °C/16 h was compared to further evaluate the filtration reduction capacity of PAASM-CaCO_3_. Table 1 shows the formulations of different drilling fluids. The main components of drilling fluids are shown in Table 4.

**Figure 9:**
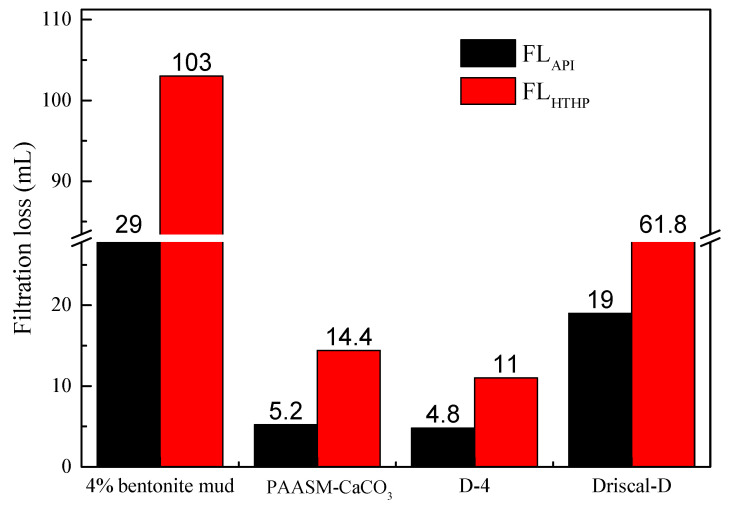
Filtration loss in based muds after aging at 200 °C.

The filtration effects of 1% PAASM-CaCO_3_, 1% D-4, and Driscal-D in the base mud are shown in Figure 9. It can be seen that the filtration of base fluid decreases to some extent after adding different filtration reducers. After aging at 200 °C, D-4 has the best filtration reduction performance. The medium pressure filtration loss is 4.8 mL, and the HTHP filtration loss is 11 mL. The filtration reduction result of PAASM-CaCO_3_ is close to that of D-4. After aging at 200 °C, the medium pressure filtration loss is 5.2 mL and the high-temperature and high-pressure filtration is 14.4 mL. After aging at 200 °C/16 h, the API filtration volume of Driscal-D is reduced by 19 mL. The test shows that PAASM-CaCO_3_ has a good filtration control effect in 4% bentonite drilling fluid.

#### 3.2.2. Effect on Rheology of WBDF

**Figure 10:**
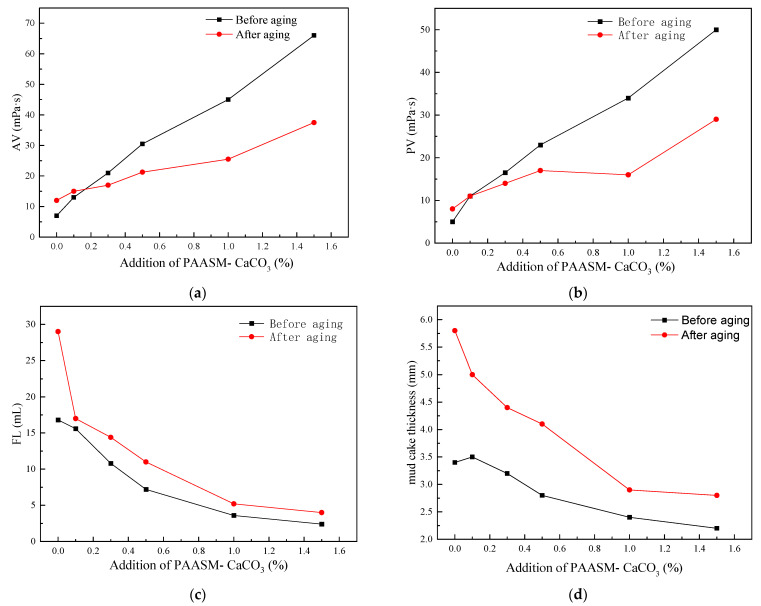
Effect of PAASM-CaCO_3_ on rheological and filtration properties of 4% bentonite mud. (**a**) Apparent viscosity; (**b**) plastic viscosity; (**c**) filtration Volume; (**d**) mud cake thickness.

In order to further study the influence of PAASM-CaCO_3_ on the rheological properties of drilling fluids, the rheological properties and filtration properties of fluids were tested with different PAASM-CaCO_3_ additions before and after aging at 200 °C/16 h. The base fluid is 400 mL deionized water + 16 g sodium montmorillonite + 0.8 g Na_2_SO_3_. The experimental results are shown in Figure 10.

It can be seen from the results that with the increase in PAASM-CaCO_3_ content in 4% bentonite mud, AV, PV, and YP gradually increase. When 1.5% PAASM-CaCO_3_ is added, the apparent viscosity of the slurry before and after aging is 66 MPa·s and 37.5 MPa·s, respectively, which indicates that PAASM-CaCO_3_ still has a good tackifying effect after aging. In addition, the yield point of the pulp before and after aging is stable under different PAASM-CaCO_3_ additions. It is also found that with the continuous addition of PAASM-CaCO_3_, the filtration loss of aging fluids decreases continuously. After aging at 200 °C/16 h, the filtration loss is 29 mL. Filtration loss is only 4 mL when 1.5% PAASM-CaCO_3_ is added. At the same time, it is not difficult to see that with the increase in PAASM-CaCO_3_, the mud cake thickness of fluid becomes thinner gradually after aging, which indicates that the mud cake quality gradually improves and starts to become thinner and tougher, which indicates that the addition of PAASM-CaCO_3_ can improve the colloidal high-temperature stability of drilling fluids.

#### 3.2.3. Comparison with Other Commonly Used Filtration Reducers

In order to further evaluate PAASM-CaCO_3_’s filtration reduction ability, the filtration control effects of PAASM-CaCO_3_ and other commonly used high-temperature-resistant filter agents before and after aging at 200 °C/16 h were compared. The results are shown in Table 5. It can be seen from the test results that the API filtration loss volume of D-4 and PAASM-CaCO_3_ are smallest after hot-rolling at 200 °C/16 h, which are 4.8 mL and 5.2 mL, respectively. The mud cake thickness of PAASM-CaCO_3_ drilling fluid after aging is similar to that of D-4 drilling fluid, which is 2.9 mm and 2.4 mm, respectively, which shows that PAASM-CaCO_3_ prevents mud cake from becoming too thick and improves the quality, indicating that the newly developed PAASM-CaCO_3_ has good filtration control and good heat resistance.

#### 3.2.4. Evaluation of Temperature Resistance

**Figure 11:**
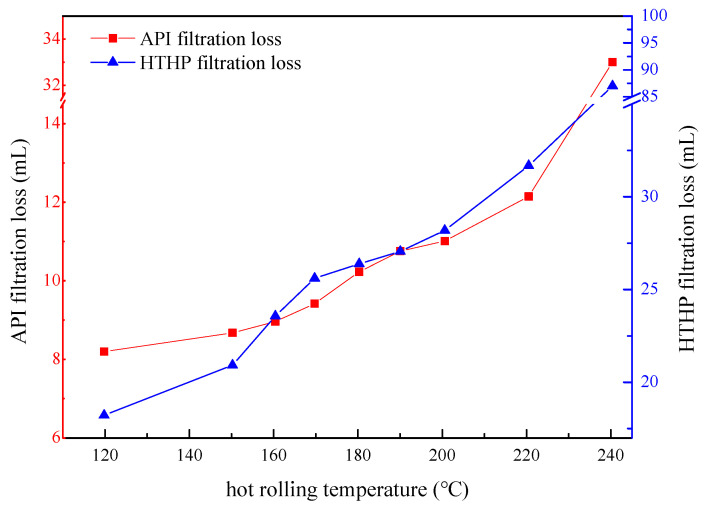
Filtration loss after aging at different temperatures.

The results of API filtration and HTHP filtration after aging at different temperatures are shown in Figure 11. It can be seen that with the increase in aging temperature, the API loss of drilling fluid gradually increases. As the temperature increases, the high-temperature and -pressure loss of drilling fluid increases rapidly at first, and then slowly and then rapidly. When the temperature is lower than 180 °C, the change in temperature has little effect on drilling fluid filtration. When the temperature exceeds 180 °C, the molecular structure of PAASM-CaCO_3_ starts to become damaged under high temperature, the movement of water molecules becomes more serious, and the hydration group of PAASM-CaCO_3_ cannot play its full role, which leads to the weakening of gel protection and the increase in filtration loss.

### 3.3. Study of Filtration Control Mechanism

#### 3.3.1. Particle Size Distribution Test

Large and small particles often coexist in drilling fluids and maintain a certain proportion. During filtration, large particles in drilling fluid can act as a bridge to support the main frame of the mud cake. Small particles play a filling role. The cage structure formed by large particles can be filled with a suitable proportion of small solid particles, thus improving the density of the filter cake and keeping the filtration performance of drilling fluids in a good state.

**Figure 12:**
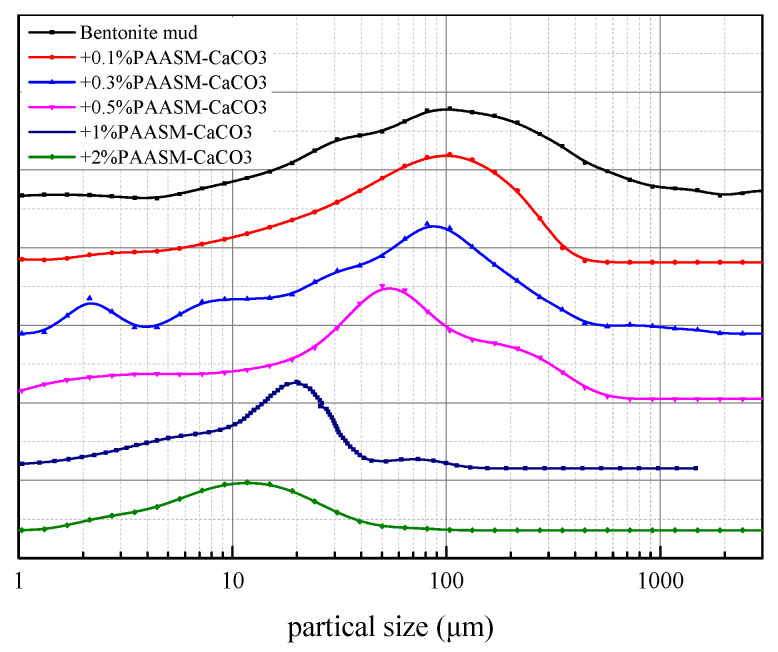
Particle size distribution after aging (in °C/16 hg).

Figure 12 and Table 6 show the test results of the particle size distribution of drilling fluids after aging in the presence of PAASM-CaCO_3_ at different concentrations. From the test results, it can be seen that the particle size distribution of basic mud is wide and multi-peaked. There are submicron, micron, and millimeter particles in the drilling fluid, and the median particle size is 28.80 μm. With the addition of PAASM-CaCO_3_, the particle size distribution of mud gradually changes from multi-peaked to single-peaked, and the particle size distribution curve moves to the left. This shows that with the increase in the PAASM-CaCO_3_, the number of small particles of micron and sub-micron size increases, and their particle size distribution becomes narrower, which makes the particles in mud more uniform. PAASM-CaCO_3_ has a strong adsorptive functional group and rigid nanostructure, which has a high adsorptive energy and surface energy. It can appear on the surface of clay particles after adding in. In addition, it improves the thickness and diffusion of hydration film and the double layer on the surface of clay particles, enhancing the water and static repulsion between particles, and at the same time, its hydrophobic structure can form a film around clay particles. Therefore, the aggregation of clay particles is restrained and clay particles are decomposed into fine particles, which is beneficial to reducing mud filtration.

**Figure 13:**
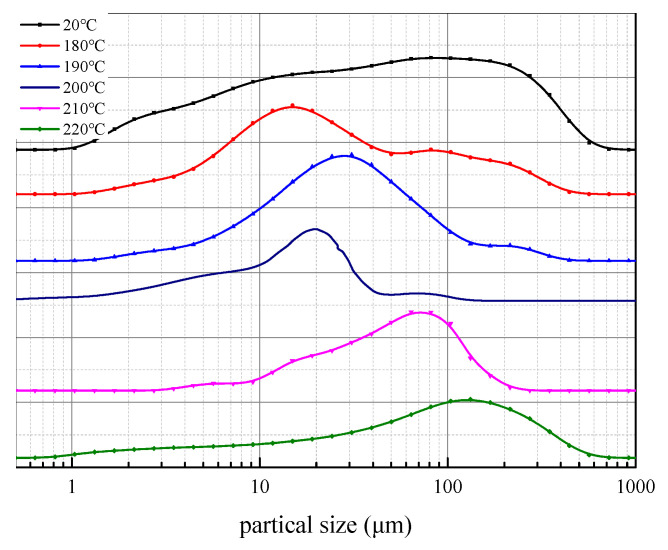
Particle size distribution curve of drilling fluids after aging.

The test results of the particle size distribution of drilling fluids before and after aging at different temperatures are shown in Figure 13 and Table 7. It can be seen that the particle size distribution of the drilling fluid is relatively wide before aging, which indicates that under this condition, the particles are conducive to the bridging action of large particles and filling action of small particles. After aging at different temperatures, the size distribution of the muds becomes narrower and the size becomes larger. It can be seen from the test results that within a certain temperature range (<210 °C), with the increase in aging temperature, the grain size distribution curve of the mud gradually moves to the left, indicating that the small particles in drilling fluid begin to increase and the large particles gradually decrease. In conclusion, PAASM-CaCO_3_ gradually plays a role in this temperature range as the temperature increases. The clay particles affected by high-temperature dehydration and aggregation are dispersed to form a relatively stable colloidal suspension system. When the temperature increases further (>220 °C), the median particle size of the mud increases, the particle size distribution curve starts to show a bi-peak distribution, and the main peak starts to move to the right, indicating that the sub-micron particles begin to increase gradually and the clay particles in the muds begin to aggregate. The reason for this may be that the ionization balance of water is promoted with increasing temperature. At this time, the active H+ in water gradually increases and the attack probability of the PAASM-CaCO_3_ molecular main chain increases greatly, which leads to the oxidation, deformation, and even decomposition of the PAASM-CaCO_3_ molecular main chain, releasing rigid nanoparticles initially encapsulated in the polymer framework and combining them. Therefore, clay particles without PAASM-CaCO_3_ protection also begin to dehydrate and aggregate at high temperatures, forming small submicron particle clusters and thinning the hydration film. The intermolecular hydration repulsion is weakened, resulting in an increase in particle size.

#### 3.3.2. Zeta Potential Test

**Figure 14:**
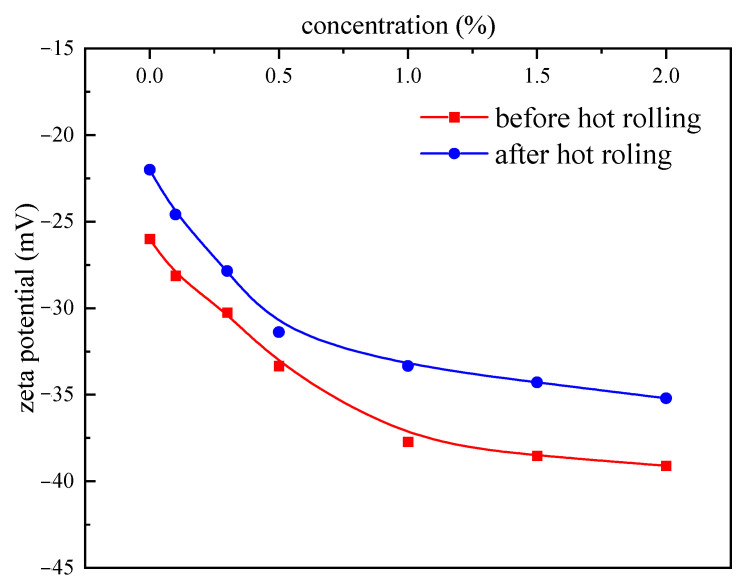
Effects of different concentrations of PAASM-CaCO_3_ on zeta potential.

The zeta potential values of PAASM-CaCO_3_ mud with different additions are shown in Figure 14. It can be seen from the test results that the zeta potential of the muds is negative throughout the experiment, which indicates that the charge properties of the surface of bentonite particles have not changed with the addition of PAASM-CaCO_3_. Before aging, with the increase in PAASM-CaCO_3_ concentration, the absolute zeta potential of the drilling fluid begins to increase, which indicates that more and more bentonite particles are adsorbed on the surface of PAASM-CaCO_3_. When the concentration of PAASM-CaCO_3_ increases to 1.5%, the absolute zeta potential of the mud does not increase significantly, indicating that under this condition, PAASM-CaCO_3_ forms saturated adsorption, continues to increase its dosage, and cannot adsorb more bentonite colloidal particles. The absolute zeta potential of the mud increases significantly with the increase in PAASM-CaCO_3_ concentration after aging at 200 °C/16 h, which indicates that the hydrate groups in PAASM-CaCO_3_ adsorb on the surface of bentonite particles at high temperature and improve the double layer thickness of diffusion electricity. The protective effect of PAASM-CaCO_3_ on colloidal particles is more obvious with the increase in PAASM-CaCO_3_ dosage. High-concentration PAASM-CaCO_3_ can be adsorbed excessively on the surface of clay particles, inhibiting the adverse effects of high-temperature dehydration. In addition, according to the zeta potential of before and after aging, it can be seen that the zeta potential of bentonite after high-temperature treatment decreases slightly compared with the absolute value before aging, but the decrease is not significant, indicating that the zeta potential source of clay particles is less affected by temperature. In the presence of different concentrations of PAASM-CaCO_3_, the absolute zeta potential of drilling fluids after aging also decreases, which indicates that some hydration and adsorption groups of PAASM-CaCO_3_ decompose at high temperature, resulting in a decrease in the amount of adsorption on the surface of clay particles.

## 4. Conclusions

A high-temperature filtration reducer with a core–shell structure (PAASM-CaCO_3_) was developed by mosaic copolymerization using AMPS, AM, St, and MA as monomers in combination with NCC. The monomers were successfully polymerized, and the weight-average relative molecular weight of the organic macromolecular backbone in the PAASM-CaCO_3_ structure was 6.98 × 10^5^. The PAASM-CaCO_3_ initial thermal decomposition temperature was high, which was about 300 °C. The newly developed PAASM-CaCO_3_ was in a spatially globular structure. PAASM-CaCO_3_ had a better resistance to high temperature. The filtration loss of drilling fluid was basically stable before and after aging at 200 °C/16 h, whose effects were near or surpassed that of Driscal-d and was on top of commonly used filtration reducers. The rigid nanoparticles were properly introduced into the PAASM-CaCO_3_, which enhanced the steric hindrance and stability of the molecular structure, and was beneficial in improving its high-temperature resistance.

## Figures and Tables

**Table 1:** Major Materials for Synthesis of PAASM-CaCO_3_.

Materials	Purity	Suppliers
Acrylamide (AM)	CP	Shanghai Sinopharm Chemical Reagent Co., Ltd., Shanghai, China
2-Acrylamido-2-Methyl Propanesulfonic Acid (AMPS)	CP	Aladdin Reagent Co., Ltd., Shanghai, China
Styrene (St)	CP	Aladdin Reagent Co., Ltd., Shanghai, China
Maleic anhydride (Ma)	AR	Shanghai Sinopharm Chemical Reagent Co., Ltd.
K_2_S_2_O_8_	AR	Shanghai Sinopharm Chemical Reagent Co., Ltd.
NaHSO_3_	AR	Shanghai Sinopharm Chemical Reagent Co., Ltd.
NaOH	AR	Shanghai Sinopharm Chemical Reagent Co., Ltd.
Span 80	AR	Shanghai Sinopharm Chemical Reagent Co., Ltd.
Tween 60	AR	Shanghai Sinopharm Chemical Reagent Co., Ltd.
N-amyl alcohol	AR	Shanghai Sinopharm Chemical Reagent Co., Ltd.
Dimethyl sulfoxide	GC	Aladdin Reagent Co., Ltd., Shanghai, China
NanoCaCO_3_ (NCC, particle size: 15 nm)	Ind	GreenSource Biotech Co., Ltd., Jinan, China

**Table 2:** Surface hydroxyl number test of NCC before and after modification.

	NCC	PAASM-CaCO_3_
V/volume (mL)	0.411	0.129
N/Hydroxyl Number	0.1506	0.0448

**Table 3:** Relative molecular weight test results of PAASM.

Weight-Average Molecular Weight (M_W_)	Number-Average Molecular Weight (Mn)	Polydispersity Coefficient (D)
697,500	284,600	2.45

**Table 4:** Preparation of drilling fluids.

Components	Amount (Concentration)			
	Base Fluid	1	2	3
Distilled water (mL)	400	400	400	400
sodium montmorillonite (g)	16	16	16	16
Na_2_CO_3_(g)	0.8	0.8	0.8	0.8
PAASM-CaCO_3_	0	4	0	0
Driscal-D	0	0	4	0
D-4	0	0	0	4

**Table 5:** Comparison of filtration control effects after aging (200 °C/16 h) of common filtration reducers.

No.	Components	Filtration Volume (mL)		MCT (mm)
		Before Aging	After Aging	
Base Fluid	4% bentonite mud	16.8	29.0	5.8
1	1#+1%PAASM-CaCO_3_	3.6	5.2	2.9
2	1#+1%Driscal-D	8.8	19.0	4.7
3	1#+1%D-4	3.2	4.8	2.4
4	1#+1%80A51	7.2	20.0	5.0
5	1#+1%JT888	4.4	18.0	4.9
6	1#+4%PJA-2	6.2	17.0	5.0
7	1#+4%FT-A	6.6	19.0	4.9
8	1#+4%SMP-I	5.2	18.0	5.0

**Table 6:** Partial size value of muds.

Concentration of PAASM-CaCO_3_/%	Particle Size/μm		
	D10	D50	D90
0	5.119	28.8	139
0.1	1.772	19.01	58.75
0.3	2.016	18.58	66.99
0.5	1.171	14.46	60.71
1	3.171	14.48	31.27
2	2.772	12.65	23.65

**Table 7:** Particle size value of muds after aging.

Aging Temperature/°C	Particle Size/μm		
	D10	D50	D90
20	1.322	12.31	72.03
180	3.824	13.7	97.59
190	4.93	16.7	57.64
200	3.171	14.48	31.27
210	4.521	16.67	40.7
220	1.796	24.17	77.94

## Data Availability

Not applicable.
